# Immunity mediates host specificity in the human hookworm *Ancylostoma ceylanicum*

**DOI:** 10.1017/S0031182023001208

**Published:** 2024-01

**Authors:** Andrea Langeland, Elise L. McKean, Damien M. O'Halloran, John M. Hawdon

**Affiliations:** 1 Department of Biological Sciences, The George Washington University, Washington, DC, USA; 2 Department of Microbiology, Immunology, and Tropical Medicine, The George Washington University, Washington, DC, USA

**Keywords:** *Ancylostoma ceylanicum*, hookworm infection, host specificity, immunodeficient model, NSG mice, permissive host

## Abstract

Hookworm infection affects millions globally, leading to chronic conditions like malnutrition and anaemia. Among the hookworm species, *Ancylostoma ceylanicum* stands out as a generalist, capable of infecting various hosts, including humans, cats, dogs and hamsters. Surprisingly, it cannot establish in mice, despite their close phylogenetic relationship to hamsters. The present study investigated the development of *A. ceylanicum* in immunodeficient NSG mice to determine the contribution of the immune system to host restriction. The infections became patent on day 19 post-infection (PI) and exhibited elevated egg production which lasted for at least 160 days PI. Infective *A. ceylanicum* larvae reared from eggs released by infected NSG mice were infectious to hamsters and capable of reproduction, indicating that the adults in the NSG mice were producing viable offspring. In contrast, *A. ceylanicum* showed limited development in outbred Swiss Webster mice. Furthermore, the closely related canine hookworm *Ancylostoma caninum* was unable to infect and develop in NSG mice, indicating that different mechanisms may determine host specificity even in closely related species. This is the first report of any hookworm species completing its life cycle in a mouse and implicate the immune system in determining host specificity in *A. ceylanicum*.

## Graphical abstract

**Figure:**
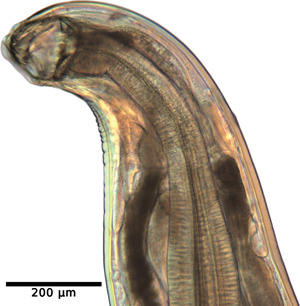

## Introduction

Hookworms, a soil-transmitted nematode, have profound health effects, impacting 500–700 million people globally (Pullan *et al.*, 2014). Adult hookworms attach to the intestinal walls and feed, causing chronic intestinal blood loss, iron deficiency anaemia and protein malnutrition (Stoltzfus *et al.*, 1997; Brooker *et al.*, 1999). Children face consequences such as stunted growth and cognitive impairments (Sakti *et al.*, 1999), while pregnant women risk adverse health outcomes, such as low birth weight and increased infant mortality (Brooker *et al.*, 2008). Given these far-reaching impacts on health, there is a pressing need to deepen our understanding of hookworm infection, to devise more effective control strategies and interventions. An intriguing area in need of research is the long-standing question of what mediates the host range of a parasite. Specifically, why can a given parasite infect certain ‘permissive hosts’ (PHs) but not other, ‘non-permissive hosts’ (NPH)? Conversely, how does an NPH prevent hookworm establishment, whereas a PH is susceptible to infection? For hookworms to successfully colonize a host, an infective larva (iL3) must recognize when it has entered a suitable environment (i.e. a PH). The host must provide the necessary nutrients for growth and reproduction, while also being vulnerable to hookworm immune evasion mechanisms, in order for the iL3 to develop and mature. Successful infection relies on detecting a PH and activating developmental programmes in response. Failure to recognize a PH results in a lost opportunity, and development in an NPH leads to death or reproductive isolation. From the host perspective, the NPH must be able to counter the hookworm's repertoire of offensive weapons, including immunomodulating molecules and virulence factors. Thus, the mechanism governing the development within the PH environment is critical to successful parasitism and represents a potential vulnerability in the life cycle.

The generalist hookworm *Ancylostoma ceylanicum* naturally parasitizes humans, cats and dogs, but it can also complete its development and reproduce in Syrian hamsters, a host relationship that is unlikely to be found in nature (Chowdhury and Schad, 1972; Ray *et al.*, 1975). Despite this, it has been used as a model for hookworm infection due to its ability to infect a small rodent and a clinical course similar to that in humans. Given the broad host range, including hamsters, it is surprising that *A. ceylanicum* cannot infect a closely related rodent like a mouse, both of which belong to the rodent family Muridae (Guénet *et al.*, 2012). Evidence suggests that a small fraction of the infecting dose (~5%) can resume development within the first 3 days in a non-immunosuppressed outbred mouse, but they invariably fail to establish or mature, and are expelled by day 8 post-infection (PI) (Ray *et al.*, 1975). Some *Ancylostoma* species, including *Ancylostoma caninum*, are capable of surviving as arrested iL3 in NPHs, a state known as hypobiosis (Lee *et al.*, 1975; Schad *et al.*, 1984; Schad, 1990). These larvae arrest in skeletal muscle of the paratenic host and remain infective for extended periods (Lee *et al.*, 1975). If a PH consumes the mouse, the hypobiotic L3 will exit arrest and infect the host as usual. Therefore, hypobiosis and paratenesis provide an additional transmission mechanism for larvae that end up in an NPH.

Failure of developing hookworms to establish in the NPH is likely due to immune-mediated factors. While adult hookworms can attach and feed in various mouse strains, leading to anaemia and weight loss, they are expelled by day 14 post-transfer, suggesting this is not due to structural intestine differences (Bungiro *et al.*, 2003). Additionally, the varied pathology across mouse strains indicates an immunological expulsion factor, consistent with known immune response differences in these strains. Secondly, hydrocortisone-treated mice become more permissive to infection with a hamster strain of *A. ceylanicum*, with some worms reaching maturity and reproducing (Ray *et al.*, 1975). Hydrocortisone suppresses immunity non-specifically and incompletely (Diehl *et al.*, 2017), making it difficult to determine which arm of the immune response restricts hookworm development. Additionally, our recent work on comparative transcriptomics from intestinal cells of a PH and NPH identified unique immune responses in the NPH (Langeland *et al.*, 2023). These immune responses were upregulated in non-permissive mice, while they were either downregulated or remained unchanged in permissive hamsters.

In this study, we leverage 2 congeneric species that differ in their host specificity to test the hypothesis that the immune system regulates permissiveness in hookworm infection. We found that *A. ceylanicum* completes its life cycle in immunodeficient NSG strain mice, which lack an effective immune system, but not in outbred Swiss Webster mice. Specifically, NSG mice lack mature T cells and B cells and possess defective cytokine signalling, resulting in a deficiency of natural killer cells (Shultz *et al.*, 2005). However, the closely related host specialist *A. caninum* failed to develop in NSG mice. Our results indicate that in the case of *A. ceylanicum*, the host immune system mediates permissiveness, but that other mechanisms restrict development of related hookworms in NPHs. These findings are the first report of a hookworm reaching maturity and producing viable offspring in a mouse, and represent a new model to investigate immune-mediated regulation of host restriction.

## Materials and methods

### Strain maintenance and preparation of infective doses

An Indian strain of *A. ceylanicum* iL3s (US National Parasite Collection No. 102954) was maintained in the Syrian golden hamster *Mesocricetus auratus* as described earlier (Langeland *et al.*, 2023). iL3s were stored in BU buffer [BUffer, 50 mm sodium hydrogen phosphate (Na_2_HPO_4_), 22 mm monopotassium phosphate (KH_2_PO_4_), 70 mm sodium chloride (NaCl), pH 6.8] (Hawdon and Schad, 1991) in a tissue culture flask at room temperature in the dark for up to 4 weeks. On the day of infection, the desired iL3 dose was obtained from the culture flask and transferred to a sterile 15 mL centrifuge tube. BU buffer was added to a total volume of 9.9 mL before 100 *μ*L concentrated hydrochloric acid (HCl) was added. The worms were incubated for 30 min at room temperature to surface sterilize (axenize) the iL3s. The infective doses were axenized for both the experimental and control groups of mice, but not for hamster infections. After axenization, 5 mL phosphate-buffered saline (PBS) buffer (10 mm Na_2_HPO_4_, 1.8 mm KH_2_PO_4_, 2.7 mm KCl, 137 mm NaCl, pH 7.0) were added and the tube was centrifuged at 2300 rpm for 5 min for washing. Using a sterile glass Pasteur pipette, the supernatant was aspirated from the tube and the washing step was repeated thrice. Finally, each 100 *μ*L dose was aliquoted into sterile 1.5 mL tubes and centrifuged at 3200 rpm for 5 min before removing 50 *μ*L supernatant to obtain 50 *μ*L infection doses for mice (100 *μ*L doses were used for hamsters). Two strains of *A. caninum* were utilized for infections: the wild-type laboratory strain (WMD) (Kitchen *et al.*, 2019) and the multi-drug-resistant BCR strain (McKean *et al.*, 2023). Both strains were maintained in beagles following previously described protocols (Schad and Page, 1982; Krepp *et al.*, 2011). Doses of *A. caninum* iL3 were prepared as described above, except that the tubes were centrifuged at 1800 rpm for 2 min.

### Animal infection and collection of small intestines

Immunodeficient ‘NSG’ mice, containing the scid mutation in the DNA repair complex protein Prkdc and the complete null allele of the interleukin-2 (IL2) receptor common gamma chain (IL2rg^null^), were obtained from the Jackson Laboratory (JAX stock #005557) (Shultz *et al.*, 2005) and donated from the Fernandes Lab at George Washington University. Animals were infected orally with ~140 surface sterilized *A. ceylanicum* iL3s in 50 *μ*L sterile PBS, followed by 50 *μ*L nuclease-free water to rinse the tube. Infected experimental animals were euthanized at 14, 21 and 28 days PI. Swiss Webster mice were used as immunocompetent controls. Control Swiss Webster mice were subjected to the same infection procedure as the NSG mice; however, the mice were euthanized at different time points: 3, 7, 11 and 15 days PI. These time points were chosen to ascertain the timing of larvae expulsion from an immunocompetent host and to investigate whether hypobiosis occurs. Immediately following euthanasia for all animals, the small intestines were removed and transferred to Petri dishes filled with PBS and incubated on a hot plate at 37°C. The small intestines were cut longitudinally to expose the intestinal epithelium, and the worms allowed to detach for up to 60 min prior to retrieval for counting. To look for arrested (hypobiotic) larvae, the Swiss Webster control mice, weighing between 22 and 24 g, were dissected, retaining only the muscle and skeleton and excluding the cranium, viscera, limbs and tail. The retained tissues were meticulously trimmed into fine pieces, resulting in an average weight of 12.20 ± 1.36 g per mouse. The cut tissue was placed in Baermann funnels overnight with PBS on days 3 and 15 PI to extract any arrested larvae from the tissues (Baermann, 1917). Infection of NSG and Swiss Webster mice was also attempted with the 2 isolates of *A. caninum*, the multi-drug-resistant BCR isolate and the wild-type WMD isolate (Kitchen *et al.*, 2019). Infections of the mouse strains with *A. caninum* were achieved in the same manner as described above for *A. ceylanicum*. The animals were infected with either 150, 250, 500 or 1000 iL3s of either isolate, and euthanized at 10, 12 or 14 days PI. As previously mentioned, the small intestine was examined, and muscle and skeleton tissues were carefully dissected and incubated in Baermann funnels for either 4 h or overnight to retrieve tissue-arrested larvae.

### Egg collection and hatching

Fecal samples were collected from infected NSG mice by placing them on wire floors. Coprocultures were established as described earlier (Langeland *et al.*, 2023) and incubated at 28°C for a minimum of 7 days, after which iL3s were recovered using a modified Baermann technique, washed and stored in 25 mL tissue culture flasks. Quantitative egg counts were determined using a modified McMaster technique (Gordon and Whitlock, 1939) at multiple time points to monitor the progression of the hookworm infection. Briefly, 2 g of fecal material were transferred to a tea strainer in a custard cup. Then, 60 mL of saturated salt solution was added and the feces were gently pushed through the strainer into the salt solution. While stirring, a pipette was used to transfer fecal solution to each chamber of the McMaster slide. The sum of the egg counts from each chamber was multiplied by 100 to obtain the total number of eggs per g (EPG) of feces. Fecal flotation was performed to confirm the presence of eggs when insufficient fecal material could be collected for quantitative McMaster process. Feces were collected in a 15 mL tube and mixed with saturated salt solution. The tube was topped off with the saturated salt solution and a coverslip was added on the top of the open tube. After 15 min, the coverslip was placed on a glass slide and examined under a microscope for eggs.

### Infection of naïve hamsters

To assess the viability of NSG-derived *A. ceylanicum*, 2 hamsters were infected with 80 NSG-derived *A. ceylanicum* iL3 larvae, and 2 others were infected with 80 iL3 of hamster-derived *A. ceylanicum* maintained in the laboratory. All hamsters were patent on day 20 PI. Prior to euthanasia on day 20 PI, quantitative fecal egg counts were conducted. Subsequently, the small intestine of each hamster was carefully extracted, placed in PBS buffer and longitudinally dissected to retrieve and enumerate adult worms.

### Data analysis and visualization

The *t.test* function from the *stats* package in R version 4.2.0 was utilized to perform a 1-sample *t*-test to determine whether there was a significant difference in the number of adults recovered at different time points (14, 21 and 28 days PI) in infected NSG mice as compared to a hypothesized mean value of zero worms. The Kruskal–Wallis rank sum test was employed for statistical analysis of data from the Swiss Webster control mice infections, using the *kruskal.test* function from the *stats* package (version 4.2.0). This test was chosen due to the non-normal distribution of the count data (number of developed worms). The Kruskal–Wallis test is especially appropriate for comparing 3 or more independent groups, even with small sample sizes, and does not assume specific data distributions. The analysis aimed to investigate significant differences in worm development across various days within the Swiss Webster control group. The *wilcox.test* function from the *stats* package (version 4.2.0) was applied to the data obtained from worm counts during the infection of naïve hamsters with NSG-derived *vs* hamster-derived *A. ceylanicum*. This non-parametric test was selected because the worm count data were not normally distributed. The Wilcoxon test is robust against such distributional irregularities and is suitable for comparing distributions of 2 independent groups. Finally, the R package *ggplot2* (version 3.3.6) was used to plot worm development and patency.

## Results

### Susceptibility of immunodeficient mice to infection with *A. ceylanicum*

**Figure 1.**
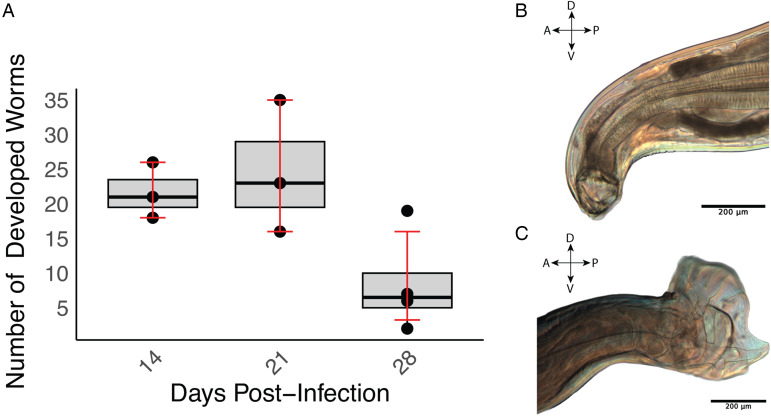
*Ancylostoma ceylanicum* successfully develop in immunodeficient NSG mice. (A) Boxplot illustrating worm recovery over time for different days of infection. The *x*-axis represents the days (14, 21 and 28) PI, and the *y*-axis represents the number of worms recovered. The boxplot displays the interquartile range (IQR), and the error bars indicate the bootstrapped confidence intervals around the means. (B) Buccal cavity, teeth and pharynx of a female adult *A. ceylanicum* at 21 days PI. (C) Copulatory bursa of a male *A. ceylanicum* at 21 days PI. For (B) and (C), the orientations are labelled: A for anterior, P for posterior, D for dorsal and V for ventral.

The developmental progression of *A. ceylanicum* in immunodeficient NSG mice was investigated at 3 time points PI (14, 21 and 28 days). The first 2 time points were comprised of 3 biological replicates (i.e. mice) each, while the final time point at 28 days encompassed 4 replicates. The average number of iL3 larvae developing at these time points were 21.7 ± 4.0, 24.7 ± 9.5 and 8.50 ± 6.5, respectively (Fig. 1A and Table 1). Statistical analysis employing a 1-sample *t*-test revealed significant development of *A. ceylanicum* (*P* = 0.0004), as compared to a null hypothesis of no development. By day 14, the iL3 larvae had developed into young adults, and on day 21 the young adults had fully matured into egg-laying adults (Fig. 1B and C). Only adults were found on day 28.

**Table 1.** *Ancylostoma ceylanicum* development in the small intestine of immunodeficient NSG mice and Swiss Webster control mice inoculated with 140 infective larvae

Days PI	Developed worms in NSG mice (mean ± s.d.)	Developed worms in Swiss Webster mice (mean ± s.d.)
3	–	3.3 ± 3.4^a^
7	–	2.3 ± 1.7^a^
11	–	0.0 ± 0.0^a^
14	21.7 ± 4.0^b^	–
15	–	0.0 ± 0.0^a^
21	24.7 ± 9.5^b^	–
28	8.50 ± 6.5^a^	–

s.d., standard deviation.

a *n* = 4 animals.

b *n* = 3 animals.

### Longevity and egg production by *A. ceylanicum* in NSG mice

**Figure 2.**
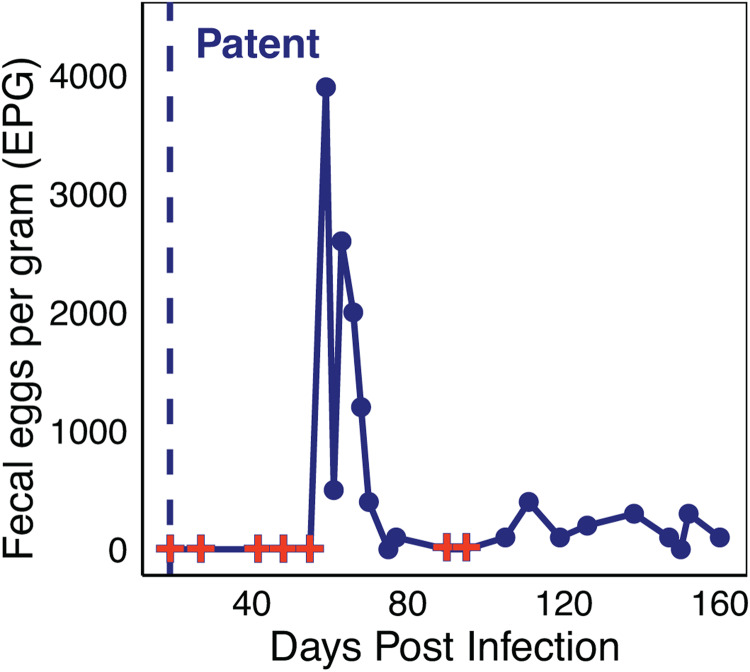
Fecal egg output following oral infection of *A. ceylanicum* in NSG mice. The plot shows the fecal egg output in EPG measured by the McMaster technique over time after oral infection of NSG mice with *A. ceylanicum*. Data points (dark blue circles) indicate the EPG at different time points. Quantitative egg counts were performed on feces collected from a cage housing 3 mice until day 77 PI and 2 mice from day 90 PI onwards. The vertical dashed line on day 19, labelled ‘Patent’, marks the onset of patency on days 18–19 PI. Red plus signs show positive fecal floatation instead of quantitative McMaster egg counts until day 59 PI and on days 90 and 95 PI.

In immunodeficient NSG mice, patency was confirmed by the presence of the eggs on days 18 and 19 in pooled feces from a cage housing 3 female mice (Fig. 2). On day 59 PI, the first quantitative egg count from the cage was 3900 EPG. Subsequently, EPG ranged from 400 to 2600 from day 61 to 70 PI, decreasing to 0 on day 75 PI. Quantitative egg counts were not conducted on days 90 and 95 PI (Fig. 2, highlighted in red), although fecal floatation confirmed the presence of eggs on those days. Moreover, during this period, 1 mouse escaped, resulting in egg counts on day 90 PI and onwards being based on 2 mice instead of the original 3. Egg counts remained consistent at 100 EPG on days 77 and 105 PI, before and after the unmeasured time period. At the final time point on day 160 PI, the EPG count was 100.

### Infectivity of NSG-derived *A. ceylanicum* larvae for a PH

To determine if iL3 derived from NSG mice were viable and infectious, 2 hamsters were infected with either NSG-derived or hamster-derived *A. ceylanicum* iL3. On day 19 and 20 PI, hamsters infected with NSG-derived and the hamster-derived *A. ceylanicum*, respectively, were patent. As the first fecal flotations were conducted on day 19 PI, it is possible that the infections were patent earlier. However, this is within the typical range of prepatent periods for *A. ceylanicum* in the lab (18–21 days). On day 20, the pooled egg counts were 4100 EPG for the NSG-derived *A. ceylanicum* strain and 1500 EPG for the laboratory *A. ceylanicum* strain. Subsequently, on day 20, the hamsters were euthanized, and examination of the small intestine revealed the presence of 22 and 40 (mean 31 ± 12.7) adult NSG-derived *A. ceylanicum* worms in each hamster, whereas each hamster infected with the hamster-derived worms had 35 and 37 (mean 36 ± 1.4) adult worms in the small intestine. A Wilcoxon rank-sum test indicated that there was no significant difference (*P* value = 1) between the ability of NSG- and hamster-derived L3 to develop in hookworm naïve hamsters.

### Infection of non-permissive mice

**Figure 3.**
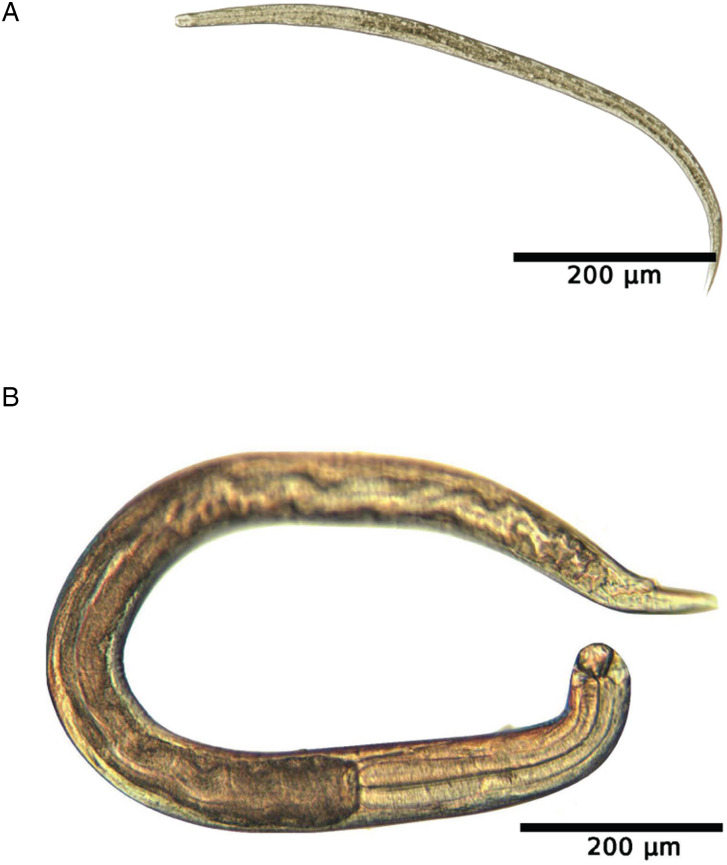
Development of *A. ceylanicum* in Swiss Webster control mice. Microscopic images depict *A. ceylanicum* recovered from the small intestine at (A) 3 days and (B) 7 days PI.

Over a 15 day period PI, *A. ceylanicum* development in the small intestine of non-permissive control mice was monitored to assess timing of expulsion and whether *A. ceylanicum* larvae arrest in an NPH. At each time point (3, 7, 11 and 15 days PI), 4 biological replicates consisting of 2 females and 2 males were used. On 11 and 15 days PI, no worms were detected (Table 1). However, on days 3 and 7 PI an average of 3.3 ± 3.4 worms and 2.3 ± 1.7 worms were observed in the small intestine, respectively (Table 1 and Fig. 3). Despite these observations, there was no significant difference in worm counts between the different days PI (*P* > 0.05). Finally, on days 3 and 15 PI, no worms, either developing or in a hypobiotic (arrested) state, were detected within other tissues.

### *Ancylostoma caninum* fails to develop in immunodeficient NSG mice

To determine whether the ability to develop in immunodeficient mice is shared by other hookworms, we infected NSG mice with the closely related canine hookworm *A. caninum*. No worms were recovered from the small intestine at 10 days PI from 3 NSG mice infected with 500 iL3 of the triple-resistant BCR isolate of *A. caninum.* Similarly, no worms of any stage were recovered from the small intestine at day 14 PI of 2 additional mice infected with 250 BCR iL3s. Infections of control mice yielded the same results. Two Swiss Webster mice were infected with 1000 iL3 of the wild-type *A. caninum* isolate WMD, and 2 mice were infected with 150 BCR iL3. The small intestines were examined at 12 and 10 days PI, respectively. In neither case any worms of any developmental stage were recovered from *A. caninum*-infected Swiss Webster mice.

## Discussion

The present study investigated the development of *A. ceylanicum* in immunodeficient NSG mice *vs* its ability to develop in an NPH. Our findings reveal the first mouse model of any strain in which *A. ceylanicum* completes its life cycle and produces viable offspring. Infections reached patency on day 19 PI, indicating that NSG mice provide a permissive environment for hookworm maturation and reproduction. The observed developmental progression of *A. ceylanicum* in NSG mice, from young adults on day 14 to fully matured, egg-laying adults on days 21 and 28, was similar to the progression of infections in the traditional golden hamster lab host. Furthermore, iL3 derived from infections in NSG mice were infectious for hamsters, producing patent infections that were not different in time to patency or worm burden from infections using hamster-derived iL3. Taken together, these data indicate that NSG mice are fully PHs for *A. ceylanicum*. Interestingly, infections in NSG mice produced egg counts of 3900 EPG at 59 days PI, which is considerably higher than the egg output from a typical hamster infection, even during the first weeks of patency (3–6 weeks PI). It should be noted that egg counts for both infected hamsters and mice were from cages housing multiple animals, so we are unable to determine the egg counts from individual infections. However, infections in NSG mice also persist longer than those in hamsters. According to our and other studies (Pan *et al.*, 2019), *A. ceylanicum* infections typically start declining on day 33 PI and are cleared by days 48–50 PI in Syrian hamsters when infected orally with 150 *A. ceylanicum* iL3s, whereas NSG mice infected orally with 140 iL3 remained patent until at least 160 days PI. The apparent increased longevity and egg output of the infections suggests that a functional immune response, rather than worm lifespan, limits egg production and infection duration in hamsters infected with *A. ceylanicum*.

Notably, *A. ceylanicum* demonstrated a limited ability to undergo development in non-permissive mice, as evidenced by the presence of a small number of developing worms on days 3 and 7 PI. Although expelled by day 11, these results suggest that a fraction of the hookworm-infective larval population retains some degree of plasticity, enabling them to respond to signals from NPHs that initiate development. These signals may differ qualitatively or quantitatively from those in PHs. This plasticity might provide an opportunity for host capture and potentially contribute to the evolution of a new host association. These results also align with previous reports of a small fraction (~5%) of the infective dose resuming development within the first 3 days in a non-immunosuppressed mouse, but ultimately failing to establish or mature, with expulsion by day 8 PI (Ray *et al.*, 1975).

The inability of the congeneric species *A. caninum* to develop in immunodeficient NSG mice is somewhat surprising, especially considering how successfully *A. ceylanicum* was able to exploit this host. The lack of a functional immune system in NSG mice suggests that, unlike in *A. ceylanicum*, the immune response of the host has little influence on the permissiveness of mice for *A. caninum*, and therefore it cannot be assumed that the immune response plays a significant role in restricting any particular parasite's host range. This is especially intriguing in that incomplete development of *A. caninum* in humans causes eosinophilic enteritis, and there have been several reports of patent *A. caninum* infections in humans (George *et al.*, 2016; Ngcamphalala *et al.*, 2019; Furtado *et al.*, 2020; Hawdon and Wise, 2021), indicating that this species is able to exploit NPHs under some circumstances. One possible explanation for the inability to exploit even a severely immunodeficient mouse is that mice and other rodents function as paratenic hosts for *A. caninum*. Developmentally arrested, or hypobiotic, larvae in the muscle of rodents remain infective for a canid host for essentially the lifespan of the rodent. The evolutionary advantage of this transmission route may exceed that of exploiting a rodent as a definitive host, and therefore there are other non-immune mechanisms that prevent development of *A. caninum* larvae in these hosts. On the other hand, little is known about the role of paratenesis and hypobiosis in the life cycle of *A. ceylanicum*. Although resumption of development by tissue-arrested larvae (‘larval leak’), seasonal variation in egg output and vertical transmission are important transmission mechanisms in *A. caninum* and the human congeneric parasite *Ancylostoma duodenale* (Stone and Peckham, 1970; Stoye and Krause, 1976; Schad and Page, 1982; Schad, 1990; Bowman, 2014; Hawdon and Wise, 2021), these phenomena have not been described in *A. ceylanicum*. If they do occur, they are unlikely to play such a central role in its life history as they do in the other species. A generalist species like *A. ceylanicum* with the ability to exploit new species relatively easily would have extensive opportunities for transmission and reproduction through the capture of new host species. Specialist species like *A. caninum* lacking mechanisms to exploit new hosts would evolve strategies that increase the chances of transmission to their limited range of definitive hosts, such as hypobiosis and paratenesis.

In any case, the findings presented herein hold significant implications for understanding the molecular determinants of host specificity in hookworms. The observed differences in development and survival between *A. ceylanicum* and *A. caninum* in NSG mice, along with the distinct immune responses detected in non-permissive mice compared to permissive hamsters (Langeland *et al.*, 2023), suggest that host-specific factors play a pivotal role in determining parasitic success. Further investigations are necessary to pinpoint the exact immune mechanism responsible for host restriction. Nonetheless, the successful development and reproduction of *A. ceylanicum* in NSG mice opens new opportunities for testing and developing anthelminthic drugs. This mouse model can serve as a valuable platform for evaluating the efficacy of novel anthelminthic compounds against hookworm infections. Additionally, the observed differences in development and survival between *A. ceylanicum* and *A. caninum* in NSG mice can aid in understanding the host-specific factors influencing parasitic success, offering insights into potential drug targets. Lastly, NSG mice provide a valuable opportunity for evaluating the efficacy of immunotherapeutic agents against parasitic infections. The severely immunosuppressed nature of NSG mice allows researchers to assess the impact of immunotherapeutic interventions more directly, as there is minimal interference from the host's immune system. This renders NSG mice an ideal model for screening and testing new immunotherapeutic agents aimed at combating parasitic diseases like hookworm infections.

In conclusion, our study indicates that unlike outbred mice, severely immunodeficient mice are PHs for the generalist hookworm *A. ceylanicum*, but not for the specialist *A. caninum*. This points to a role of the immune system in determining host specificity in some, but not all, host–parasite associations. The findings shed light on the intricacies of host–parasite interactions and the potential plasticity of hookworm infective larvae. Understanding the molecular basis of host specificity will be useful in devising effective control strategies and interventions to combat hookworm infections and alleviate the burden they impose on global health.

## Data Availability

Data supporting the findings of this study are available in this article.
